# Reference Intervals for Serum Thyroid-Stimulating Hormone Based on a Recent Nationwide Cross-Sectional Study and Meta-Analysis

**DOI:** 10.3389/fendo.2021.660277

**Published:** 2021-06-01

**Authors:** Xichang Wang, Yongze Li, Xiaodan Zhai, Haoyu Wang, Fan Zhang, Xiaotong Gao, Shengyu Liu, Weiping Teng, Zhongyan Shan

**Affiliations:** ^1^ Department of Endocrinology and Metabolism and the institute of Endocrinology, The NHC Key Laboratory of Diagnosis and Treatment of Thyroid Diseases, The First Hospital of China Medical University, Shenyang, China; ^2^ Department of Endocrinology and Metabolism, Shengjing Affiliated Hospital of China Medical University, Shenyang, China

**Keywords:** TSH, reference interval, cross-sectional study, meta-analysis, iodine status, ethnicity

## Abstract

**Objective:**

The aim of our study was to compare the reference intervals (RIs) [median (2.5^th^-97.5^th^ percentiles)] for thyroid-stimulating hormone (TSH) between subgroups stratified by ethnicity and iodine status in a global context.

**Design and Methods:**

Primary data were derived from a recently published cross-sectional study in mainland China. Secondary data were obtained from online databases. The RIs for TSH were calculated in the reference population according to the National Academy of Clinical Biochemistry (NACB) standard and in the disease-free population. A meta-analysis of ethnicity- and iodine status-specific TSH RIs was performed.

**Results:**

The primary data showed that the TSH RI (mU/L) in the disease-free population was 2.33 (0.67, 7.87), which is wider than the published RI [2.28 (0.74, 7.04)] in the reference population. The meta-analysis showed that whether in the reference or disease-free population, the RIs in Yellows were much higher than those in Caucasians. In the reference population, the median and 2.5^th^ percentile in the iodine-sufficient subgroup were both lower than the iodine-deficient or more-than-adequate subgroup, while the 97.5^th^ percentile showed a positive trend with increasing sufficiency of iodine. However, in the disease-free population, the iodine-sufficient subgroup had a lower median and 97.5^th^ percentile but higher 2.5^th^ percentile than the iodine-deficient subgroup.

**Conclusion:**

Yellows have a higher TSH RI than Caucasians. In the reference population, both the median and 2.5^th^ percentile TSH in the iodine-sufficient population were the lowest among the different iodine status subgroups, while the 97.5^th^ percentile of TSH showed an upward trend with increasing iodine sufficiency.

## Introduction

Subclinical hypothyroidism (SCH) is characterized by elevated serum thyroid-stimulating hormone (TSH) and normal thyroxine levels and is the most common form of thyroid dysfunction (1). According to the third National Health and Nutrition Examination Survey (NHANES III), approximately 13 million people in the US suffer from SCH, with a prevalence of 4.3% (2). The latest cross-sectional study in mainland China showed that the prevalence of SCH is 12.93% (3). Although the current prevalence is slightly lower than that in 2010 (16.7%), it is still much higher than those in other countries (4). Many studies have shown that SCH is closely related to the occurrence and progression of hypertension, dyslipidemia, coronary heart disease and other diseases (5) and is even related to the risk of cardiovascular and all-cause mortality (6). SCH with TSH levels higher than 10 mU/L, especially when coupled with positivity for thyroid peroxidase antibody (TPOAb), is likely to progress to overt hypothyroidism (7). Therefore, SCH has increasingly become a focus of research in the field of endocrinology.

Compared with patients with overt hypothyroidism, most SCH patients have no obvious symptoms, and their diagnosis is mainly based on laboratory tests. Serum TSH is the most sensitive indicator for the diagnosis. Therefore, the reference interval (RI) for serum TSH is the key to determining the prevalence of SCH. A number of previous studies have confirmed that the detected level of TSH is affected by many factors, such as age, sex, lifestyle, obesity and pregnancy (8–12); in addition, ethnicity and iodine status are important factors. As early as 30 years ago, several studies showed that Black populations generally have lower TSH levels than Caucasians (13–15), and the NHANES III study also showed that the median TSH in the reference Caucasians (1.43 mU/L) was higher than that in the Black (1.19 mU/L) and Mexican (1.36 mU/L) subgroups. A recent genome-wide association study (GWAS) also reported the discovery of 74 loci that are significantly associated with TSH levels, which confirms the impact of genetic background (16). In addition, iodine status also plays an important role. The Thyroid Disease, Iodine Nutrition and Diabetes Epidemiology (TIDE) study showed the current upper limit of TSH (7.04 mU/L) in the reference population 20 years after the implementation of the Universal Salt Iodization (USI) policy in mainland China (12). Moreover, the median and 97.5^th^ percentile of TSH had a significant increasing trend with increasing iodine levels in the TIDE study. Another survey in three regions with different iodine statuses (deficiency, more than adequate iodine and excess) revealed an iodine-related increase in the TSH RI in the reference population, which could also explain the differences found in various countries (17).

The current recommendation is to use the standard proposed by the National Academy of Clinical Biochemistry (NACB) to establish the TSH RI (18), which is as follows: to select at least 120 individuals 1) who have no personal or family history of thyroid disease, 2) who are negative for thyroid antibodies, 3) who have no visible or palpable goiter and 4) who have not received any treatments affecting thyroid function (except estrogen). The 2.5^th^ and 97.5^th^ percentiles of the log-transformed TSH are identified as the RI for TSH. However, in many studies, it is not possible to determine antibody positivity or inquire about specific individual details; thus, the inclusion and exclusion criteria for the disease-free population in the NHANES III study have been adopted. Specifically, the disease-free population includes subjects without reported thyroid disease, goiter, or use of treatments affecting thyroid function (2). Although the RI for TSH calculated based on the disease-free standard is not as accurate as the NACB standard, several large-scale studies based on this method have provided valuable information about the specific RIs for TSH in different regions (19, 20).

The present study further obtained the TSH RI in the disease-free population (N=71158) of the TIDE study to supplement the results obtained by Zhao et al. (12). Based on the current TSH RIs obtained with the two standards, the relative descent rate (RDR) of TSH in each study was further subjected to meta-analysis to determine and compare the RIs for TSH in subjects with different ethnicities and iodine statuses.

## Materials and Methods

### RI for TSH in the Disease-Free Population in the TIDE Study

The implementation of the TIDE study has been previously described in several articles (3, 12, 21). Briefly, this nationwide cross-sectional study was conducted with a multistage, stratified sampling method from urban and rural areas in 31 provinces/cities in mainland China. The inclusion criteria for the subjects were as follows: aged 18 years or older, no use of iodine drugs or contrasts within three months, and not pregnant. In view of the fact that the effect of iodine status on thyroid function is a long-term process, to ensure that the subjects’ iodine nutrition level did not suddenly change during the survey, all subjects must have lived in the community for at least five years. All subjects were asked to complete informed consent forms and questionnaires collecting their demographic information, personal and family histories of thyroid disease and medication history. Fasting blood and urine samples were collected. Afterwards, serum and urine samples were transported at -20°C to the central laboratory in Shenyang, China.

The urine iodine concentration (UIC) was measured with inductively coupled plasma mass spectrometry (ICP-MS) (Agilent 7700x, Agilent Technologies, US). The target values for the standards were 70.8 ± 9.0 μg/L, 143 ± 10 μg/L and 224 ± 14 μg/L, with intra-assay coefficients of variation (CVs) of 2.7%, 1.4% and 2.3% and interassay CVs of 2.3%, 2.5% and 2.4%, respectively. Serum TSH was measured by electrochemiluminescence immunoassay on a Cobas 601 analyzer (Roche Diagnostic, Switzerland). The RI for TSH provided by the manufacturer was 0.27-4.2 mU/L. The functional sensitivity of the TSH assay was 0.014 μU/mL. The intra-assay CV and interassay CV were 1.1-6.3% and 1.9-9.5%, respectively.

### Literature Search Strategy

A literature search was performed by two independent researchers in October 2020 in the PubMed, EMBASE and Cochrane Library databases and Google Scholar. A third researcher adjudicated any differences of opinion. The keywords “thyrotropin”, “thyroid-stimulating hormone”, “TSH”, “reference range”, and “reference interval” were used. Titles and abstracts were selected in the searching catalog. To avoid missing relevant studies, the references of each study and relevant reviews were also carefully examined.

### Inclusion and Exclusion Criteria for the Studies

Articles were selected if they met all of the following criteria: 1) they had clear inclusion and exclusion criteria; 2) the inclusion criteria were based either on the NACB or the disease-free population criteria; 3) all subjects were at least 17 years old; 4) they were the most detailed article out of any duplicate articles pertaining to the same study; and 5) they were conducted in regions with distinct ethnic groups.

**Figure 1:**
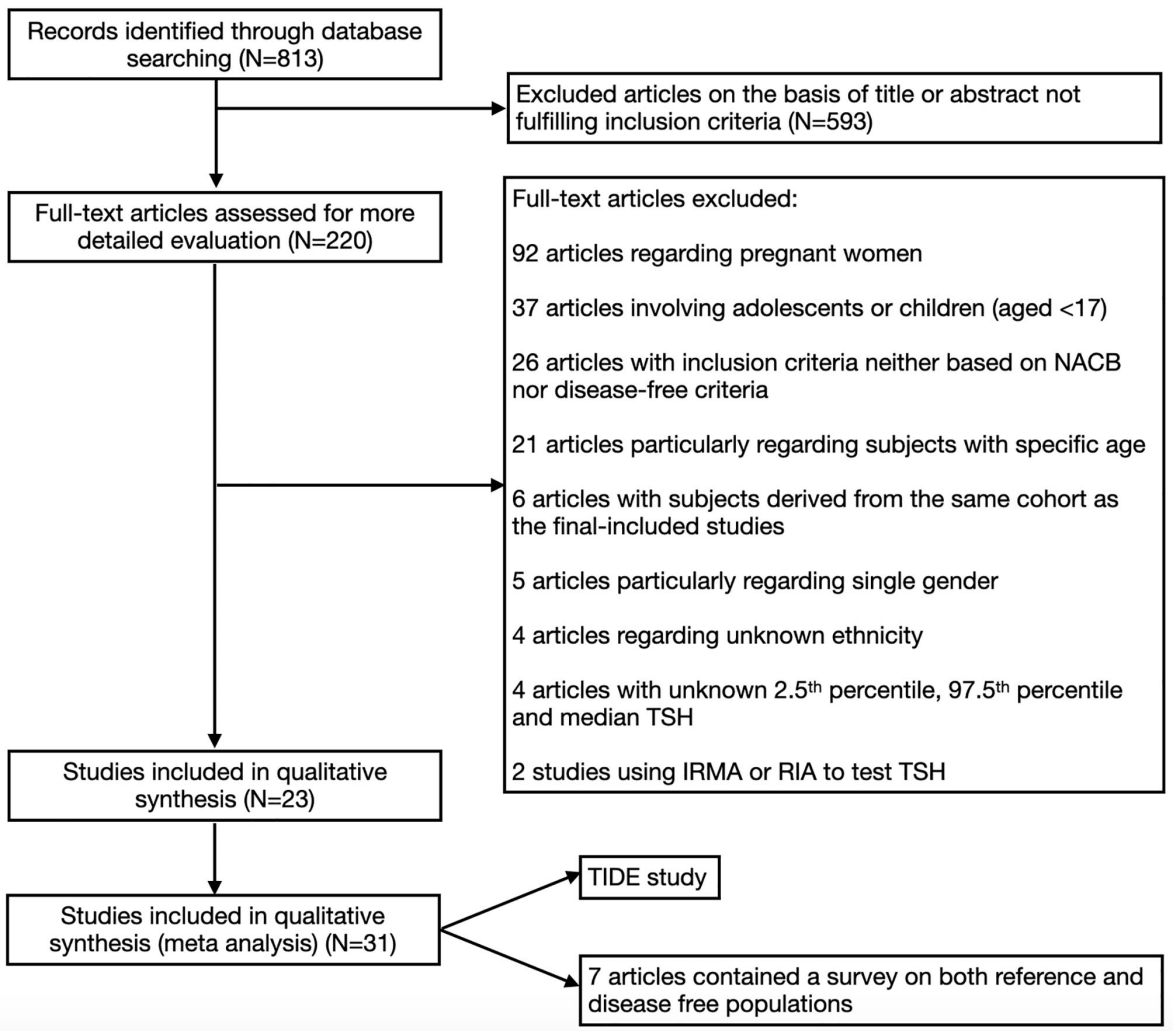
Flow diagram for the literature process.

Articles were excluded if they met one or more of the following criteria: 1) they were carried out among pregnant women; 2) they only included subjects within a specific age range (such as ≥60, 45-65, etc.); 3) they only included participants of one sex; 4) an immunoradiometric assay (IRMA) or radioimmunoassay (RIA) was used to test the TSH level; or 5) the median, 2.5^th^ percentile and 97.5^th^ percentile for TSH were all unavailable. The overall process of selecting the studies is shown in **Figure 1**.

### Data Extraction and Calculation

Detailed information was extracted from all included studies, including the first author, publication year, country, iodine status, sample size, sex ratio, age range, manufacturer, RI (median with 2.5^th^ or 97.5^th^ percentile), and RDRs of the RIs based on the data from the TIDE study.

RDRs were calculated according to the formula in the meta-analysis published by Gao et al. (22), which is as follows (the RDRs of the 2.5^th^ and 97.5^th^ percentiles of TSH were calculated in the same way):

$$RDR\;of\;median=\frac{median\;in\;the\;TIDE\;study-median\;in\;the\;corresponding\;study}{median\;in\;the\;TIDE\;study}\times 100\%$$

### Quality Assessment

The quality of the included studies was independently assessed by two investigators with the Newcastle-Ottawa Scale (NOS) for non-randomized controlled trials (RCTs) (23). The maximum score was nine stars, and the minimum was zero. Only studies with at least five stars were finally included.

### Statistical Analysis

The primary data from the TIDE study were input into Statistical Package for the Social version 25 (SPSS Inc., Chicago, IL, USA). Since the TSH level does not have a Gaussian distribution, a log-transformation was performed.

The pooled RDRs and 95% CIs were subjected to meta-analysis to estimate the RIs for TSH in various subgroups. The heterogeneity among the studies was estimated with the chi-squared-based Q test and the *I^2^* test, and 25%, 50% and 75% were considered low, moderate and high levels of heterogeneity, respectively. If the heterogeneity test showed moderate or high levels, a random-effects model was adopted; otherwise, a fixed-effects model was used. Sensitivity analysis was conducted by the sequential exclusion of each study. The meta-analysis was performed using *Review Manager (RevMan) [computer program] (Version 5.4.1, The Cochrane Collaboration, 2020).*

## Results

### RI for TSH in the Disease-Free Population in the TIDE Study

As stated in a previous study^3^, the initial number of participants in the TIDE study was 80,937. After excluding subjects with incomplete information on age, sex, or thyroid function, 78,470 remained. Then, we further excluded subjects with a personal history of thyroid disease, those who had a goiter and those who had taken medications that affect thyroid function, yielding 71158 disease-free subjects. The overall RI (median and 2.5^th^-97.5^th^ percentile) for TSH (mU/L) in the disease-free population was 2.33 (0.67, 7.87).

### Characteristics of Included Studies

As shown in **Figure 1**, a total of 813 articles were initially identified by our screening process. After examining the titles and abstracts of each article, 220 remained. Then, we excluded articles that did not meet the requirements by examining the full texts. Twenty-three articles were included, of which 7 articles contained a survey on both reference and disease-free populations. In addition, the TIDE study was also included in the present meta-analysis; thus, 31 studies were included (12, 19, 20, 24–44).

A detailed description of the abovementioned studies is presented in **Table 1**.

**Table 1:** Characteristics of the selected studies.

Reference population								
Author	Published year	Country	IS	Sample size	Gender (M: F)	Age	Manufacturer	RI (mU/L)
Ren	2020	China	MTA	2020	812:1208	18-60	Siemens	2.20 (0.62, 5.23)
Cai	2016	China	MTA	717	330:387	20-85	Siemens	1.65 (0.43, 5.51)
Kim	2015	Korea	–	7686	5683:2003	20-79	DiaSorin S.p.A.	0.72-6.80
Amouzegar	2013	Iran	S	2199	953:1246	≥20	Roche	1.46 (0.32, 5.06)
Marwaha	2013	India	MTA	1916	916:1000	18-86	Roche	Male: median=1.60; Female: median=1.74
Chan	2011	HK, China	–	157	71:86	21-71	Roche	1.56 (0.68, 3.70)
Li	2011	China	S	2118	850:1268	20-85	DPC	0.46-5.19
Quinn	2009	HK, China	S	414	217:197	20-63	Abbott	1.27 (0.37, 2.82)
Takeda	2009	Japan	–	857	441:416	20-86	Roche	1.65 (0.51, 4.57)
Hickman	2017	Australia	–	1177	630:547	≥18	Abbott	0.43-3.28
O’Leary	2006	Australia	S	2026	1047:979	17-90	DPC	1.25 (0.4, 4.0)
Clerico	2018	Italy	D	120	85:35	18-67	Beckman	1.456 (0.437, 2.89)
Tozzoli	2018	Italy	D	240	120:120	20-65	Siemens	1.66 (0.56, 3.27)
Azaric	2017	Bosnia and Herzegovina	S	224	73:151	19-70	Roche	1.96 (0.65, 5.39)
Ittermann	2015	Germany	S	1596	979:617	20-80	Siemens	0.49-3.29
d’Herbomez	2005	Germany France and Italy	D	710	412:298	18-65	Beckman	1.26 (0.35, 3.48)
Kratzsch	2005	Germany	–	453	279:174	18-68	Roche	1.36 (0.40, 3.77)
Volzke	2005	Germany	S	1488	825:663	20-79	Byk Sangtec	0.25-2.12
Jensen	2004	Denmark	D	987	527:460	17-66	IFMA kit	0.58-4.07
**Disease-free population**								
**Author**	**Published year**	**Country**	**IS**	**Sample size**	**Gender (M: F)**	**Age**	**Manufacturer**	**RI**
Cai	2016	China	MTA	1181	459:722	20-85	Siemens	Median=1.71
Kim	2015	Korea	–	19465	11616:7849	20-79	DiaSorin S.p.A.	0.62-7.70
Chan	2011	HK, China	–	212	88:124	21-71	Roche	1.58 (0.65, 3.86)
Takeda	2009	Japan	–	1007	487:520	20-86	Roche	1.77 (0.39, 4.99)
Raverot	2020	France	–	156025	65487:90538	20-108	Abbott	Male: median=1.30; Female: median=1.30
Clerico	2018	Italy	D	137179	Not mentioned	≥18	Beckman	1.73 (0.36, 5.28)
Kutluturk	2014	Turkey	D	408	268:140	≥18	Roche	1.40 (0.38, 4.22)
Vadiveloo	2013	UK	–	153127	62368:90759	≥18	Roche	Male: median=1.72; Female: median=1.70
Arachchige	2012	Australia	S	141049	Not mentioned	>20	Siemens	Range of median TSH: 1.47-1.91; range of 2.5^th^ percentile: 0.46-0.55; range of 97.5^th^ percentile: 3.62-5.64
Kratzsch	2005	Germany	–	870	445:425	18-68	Roche	1.31 (0.30, 3.63)
Volzke	2005	Germany	S	3915	2032:1883	20-79	Byk Sangtec	Range of median TSH: 0.53-0.87

IS, iodine status; D, iodine deficient; S, iodine sufficient; MTA, more than adequate; E, excess; RI, reference interval.

The RIs for TSH are presented as median and 2.5^th^-97.5^th^ percentile interval (mU/L), some of the results are not shown if absent.

The disease-free population has excluded participants who reported personal history of thyroid disease, goiter, or receiving treatments affecting thyroid function. The screening criteria of the reference population is defined by the National Academy of Clinical Biochemistry (NACB) guidelines, with or without ultrasonography examination.

### Ethnicity-Specific RIs for TSH in the Reference Subgroups

**Figure 2:**
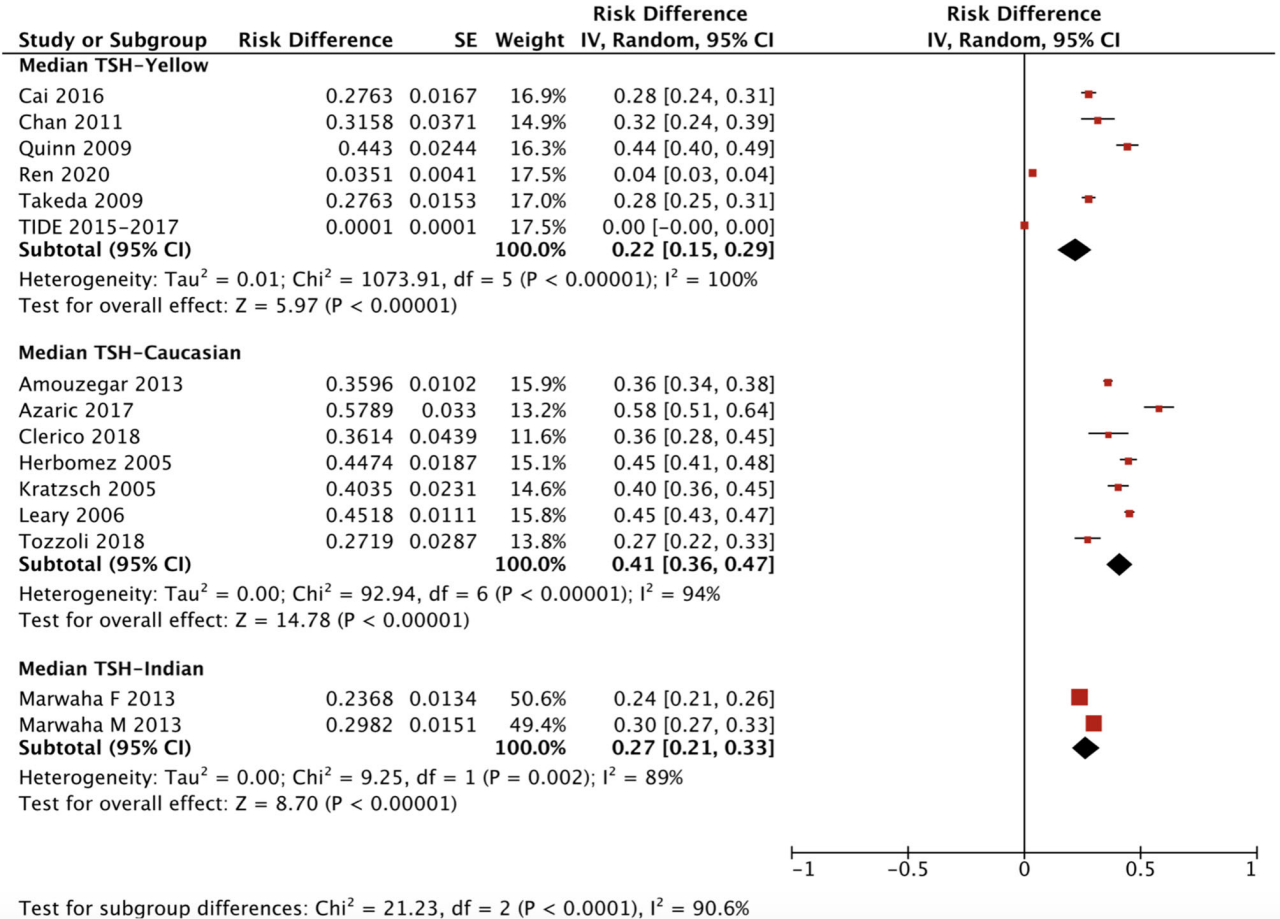
Ethnicity standardized forest plots of the pooled relative descent rate (%) of median TSH (50^th^ percentile) in NACB populations. F, female; M, male.

**Figure 3:**
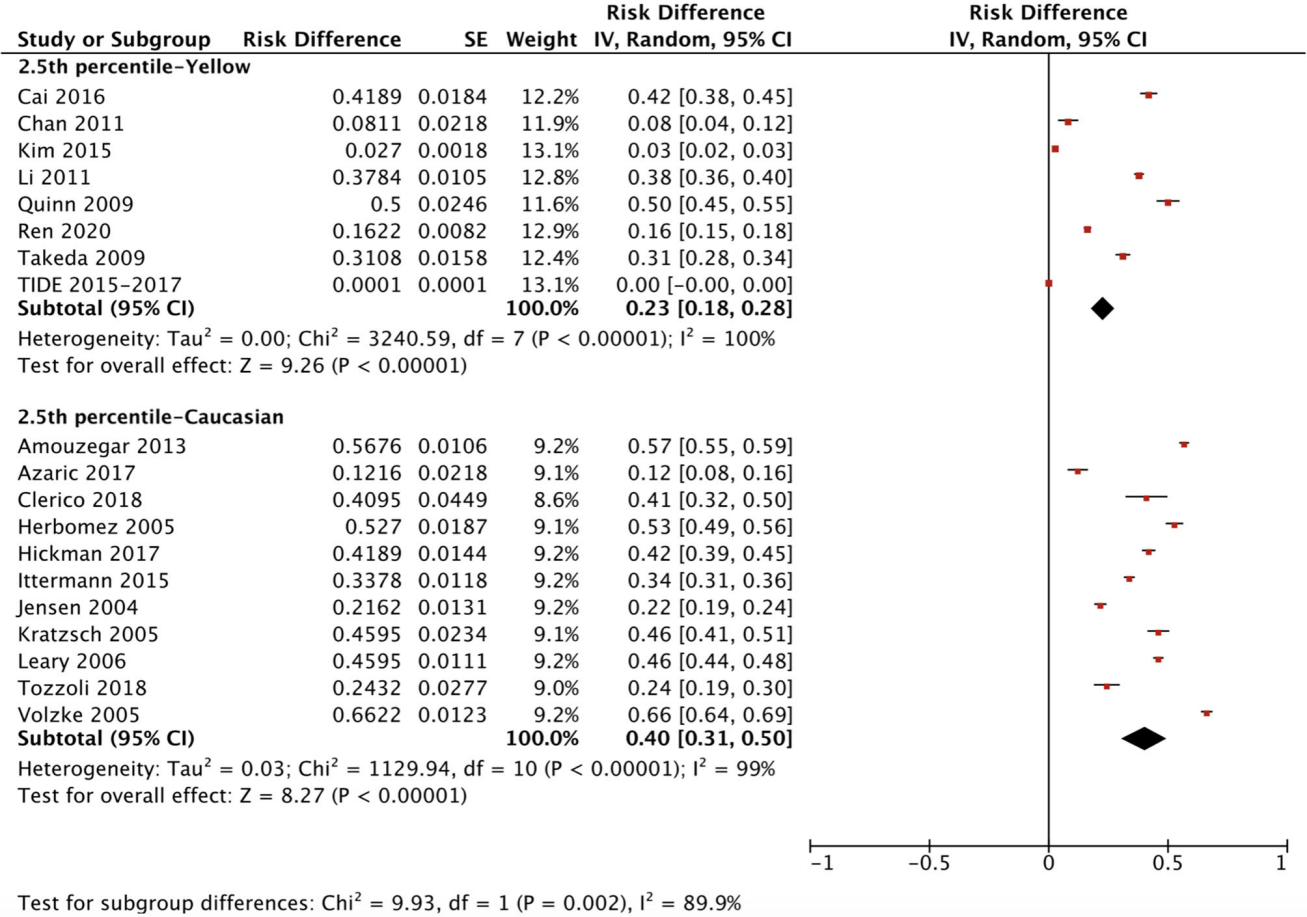
Ethnicity standardized forest plots of the pooled relative descent rate (%) of TSH lower limits (2.5^th^ percentile) in NACB populations.

**Figure 4:**
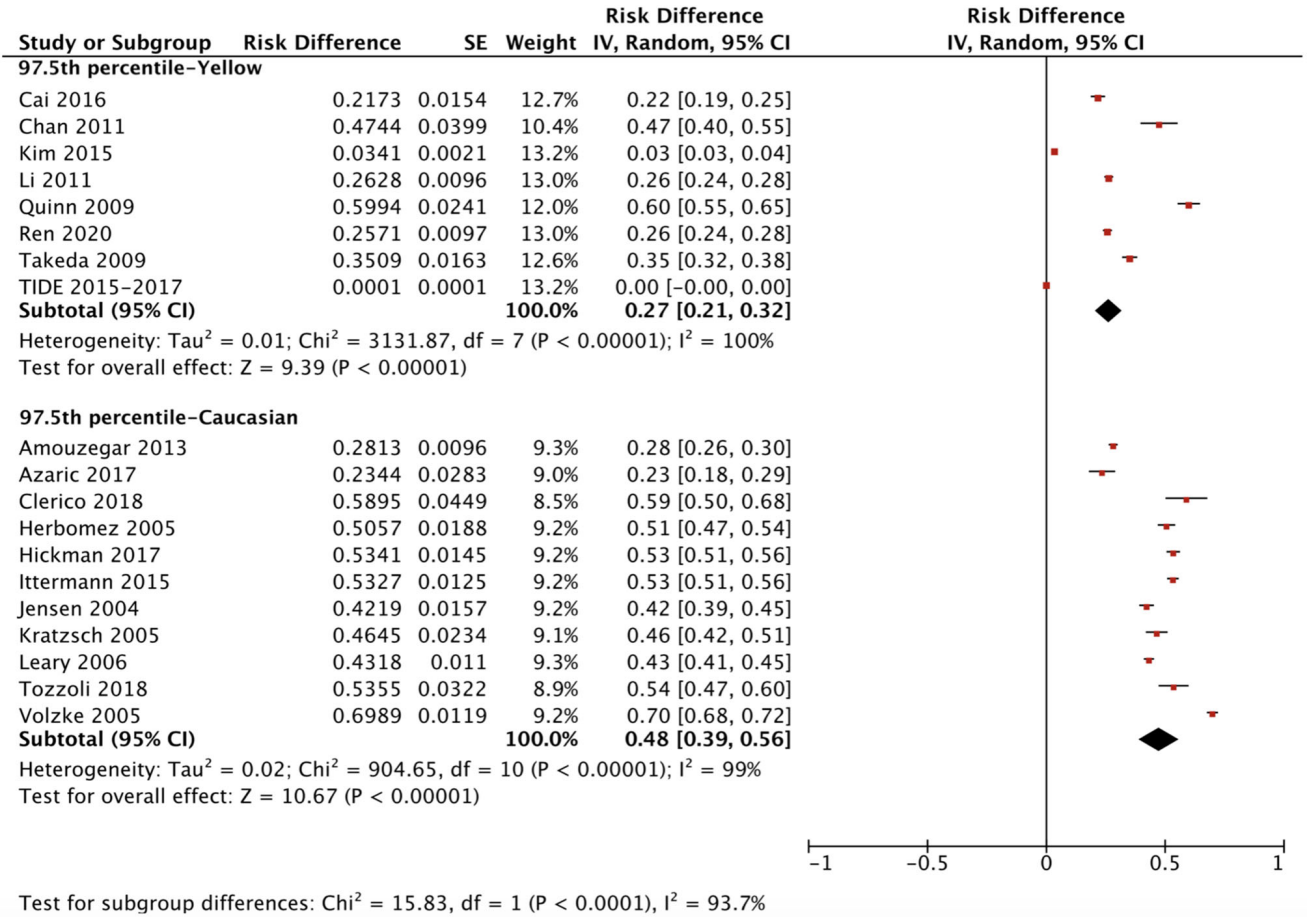
Ethnicity standardized forest plots of the pooled relative descent rate (%) of TSH upper limits (97.5^th^ percentile) in NACB populations.

The ethnicity-specific RDR of median and 2.5^th^-97.5^th^ percentile of TSH in the reference population were subjected to meta-analysis. As shown in **Figure 2**, the corresponding 95% CI of the RDR of the median TSH in Yellows does not overlap with that in Caucasians; thus, we considered the RDR in Yellows [0.22 (0.15, 0.29)] to be much lower than that in Caucasians [0.41 (0.36, 0.47)], while the RDR of the median TSH is slightly lower in Yellows than in Indians [0.27 (0.21, 0.33)]. A similar difference can be observed in **Figure 3**, namely, Yellows have a much lower RDR of the 2.5^th^ percentile [0.23 (0.18, 0.28)] than Caucasians [0.40 (0.31, 0.50)]. Similarly, the RDR of the 97.5^th^ percentile of TSH was also much lower in Yellows [0.27 (0.21, 0.32)] than in Caucasians [0.48 (0.39, 0.56)] (**Figure 4**).

After calculating the RIs and RDRs, we inferred the ethnicity-specific median and 2.5^th^-97.5^th^ percentile of TSH in the reference population. As shown in **Table 2**, the median and 2.5^th^ and 97.5^th^ percentiles (mU/L) of the Yellows in the reference population [1.78 (0.57, 5.14)] are all much higher than those in the Caucasians in the reference population [1.35 (0.44, 3.66)]. In addition, the Indians in the reference population had a median TSH of 1.68 mU/L, which is similar to but slightly lower than that in the Yellows in the reference population.

**Table 2:** The reference interval of TSH stratified by ethnicity and iodine status in the reference and disease-free population.

Subgroups	Reference population		Disease-free population	
	Number of studies	RI (mU/L)	Number of studies	RI (mU/L)
Ethnicity				
Yellow	8	1.78 (0.57, 5.14)	5	1.84 (0.58, 6.69)
Caucasian	11	1.35 (0.44, 3.66)	7	1.30 (0.38, 4.49)
Indian	1	Median=1.68	–	-
Iodine status				
Deficient	4	1.46 (0.48, 3.45)	2	1.56 (0.36, 4.80)
Sufficient	8	1.44 (0.46, 4.36)	3	1.28 (0.52, 4.49)
More than adequate	3	1.80 (0.53, 5.35)	1	Median=1.71

The reference interval of TSH are presented as median and 2.5^th^-97.5^th^ percentile after a calculation with relative descent rate.

### Iodine Status-Specific RIs for TSH in Reference Subgroups

**Figure 5:**
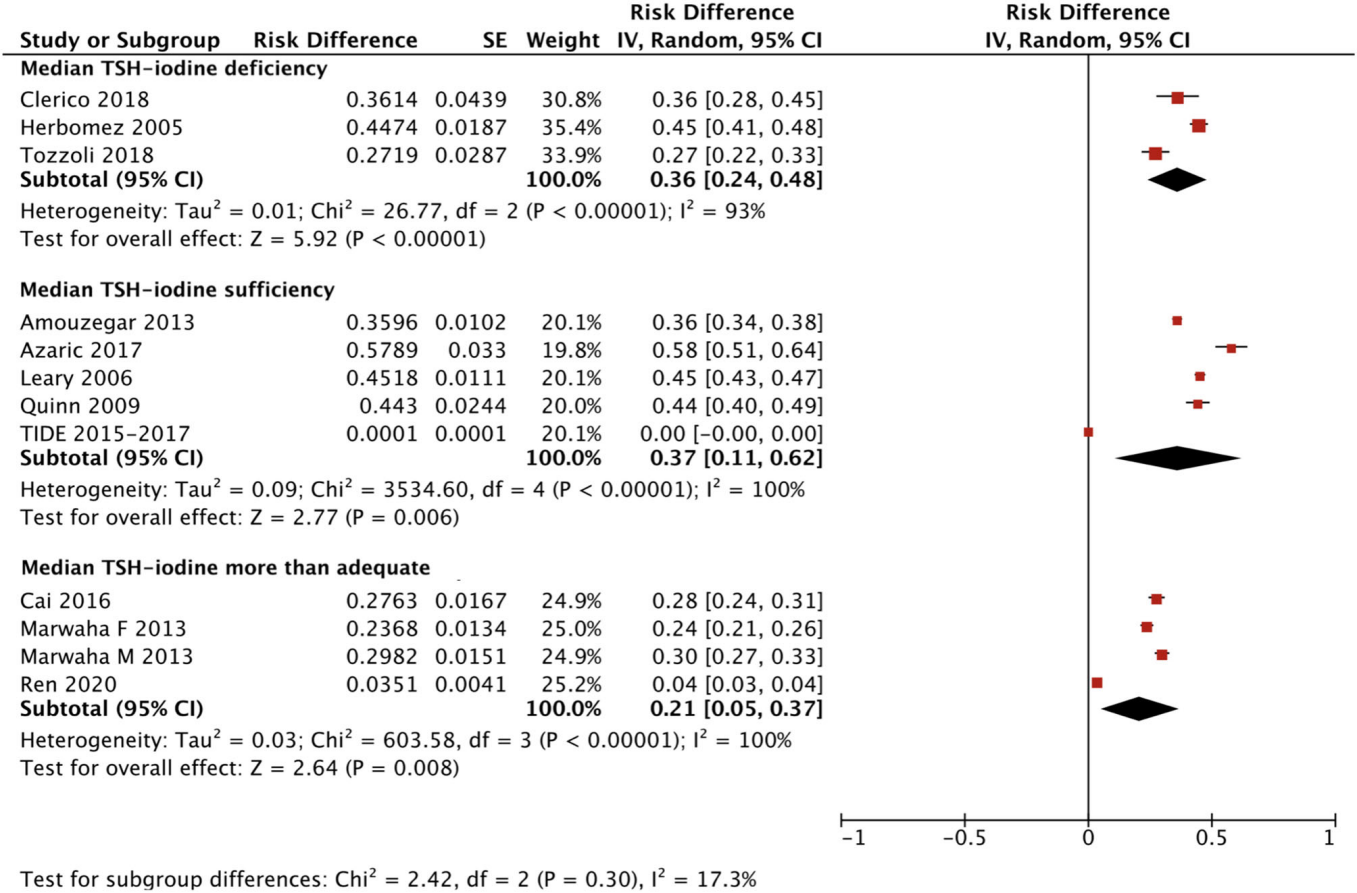
Iodine status standardized forest plots of the pooled relative descent rate (%) of median TSH (50^th^ percentile) in NACB populations.

**Figure 6:**
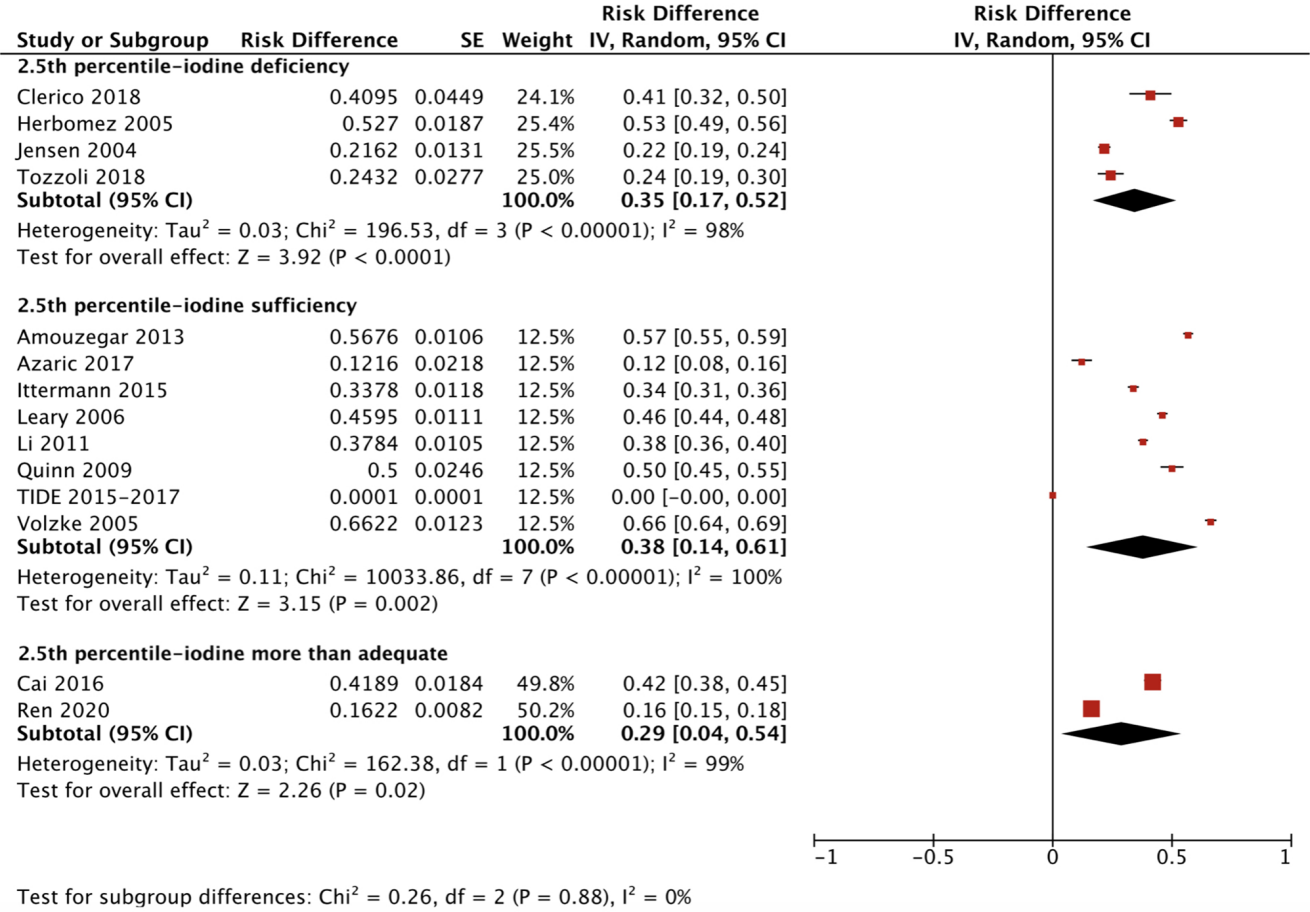
Iodine status standardized forest plots of the pooled relative descent rate (%) of TSH lower limits (2.5^th^ percentile) in NACB populations.

**Figure 7:**
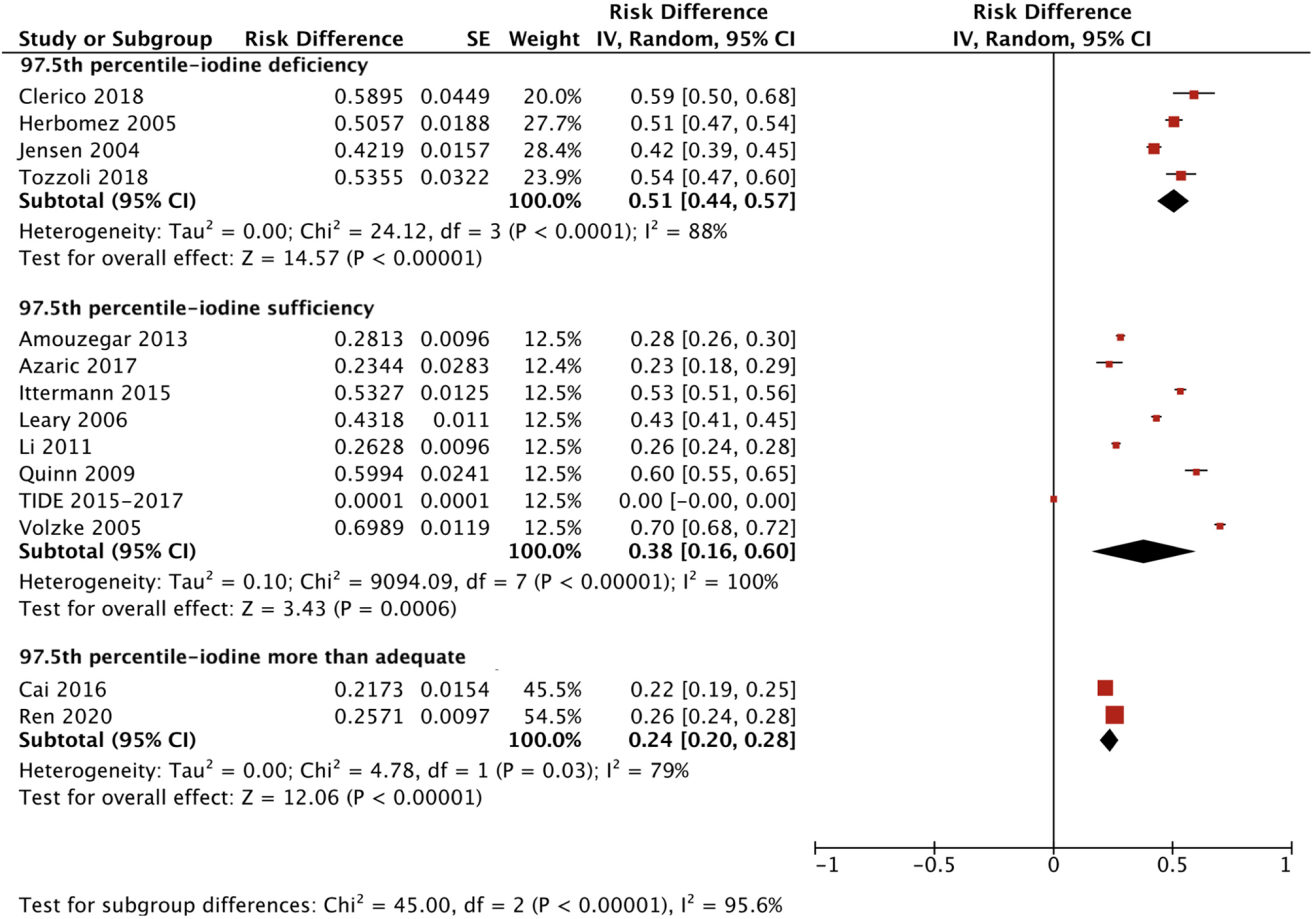
Iodine status standardized forest plots of the pooled relative descent rate (%) of TSH upper limits (97.5^th^ percentile) in NACB populations.

We applied a similar method to analyze the specific RIs of TSH in the reference population with various iodine statuses (iodine deficiency, iodine sufficiency and more than adequate iodine). The forest plots in **Figures 5**, **6** and **7** show the iodine status-specific RDRs of median, 2.5^th^ and 97.5^th^ percentiles of TSH, respectively. As shown in the three forest plots, the subgroup 95% CIs of the RDRs overlap with each other, whether they are for the median, 2.5^th^ or 97.5^th^ percentile of TSH. Therefore, we concluded that the RDR of TSH may not differ as much according to iodine status as it does among various ethnicities.

The medians and 2.5^th^-97.5^th^ percentiles in each iodine status subgroup are presented in **Table 2**. As shown in **Table 2**, the median (1.44 mU/L) and 2.5^th^ percentile (0.46 mU/L) of TSH in the iodine-sufficient reference subgroup were lower than those in the iodine-deficient (median: 1.46 mU/L; 2.5^th^ percentile: 0.48 mU/L) and more than adequate iodine reference subgroups (median: 1.80 mU/L; 2.5^th^ percentile: 0.53 mU/L), of which the more than adequate iodine subgroup had the highest level. In addition, the 97.5^th^ percentile of TSH showed an increasing trend (3.45 mU/L, 4.36 mU/L and 5.35 mU/L) from iodine deficient to sufficient and more than adequate, respectively.

### Ethnicity-Specific RIs of TSH in the Disease-Free Subgroups

Similarly, ethnicity-specific and iodine status-specific RDRs for the TSH level (median and 2.5^th^-97.5^th^ percentile) in the disease-free subgroups were also subjected to meta-analysis as above. As shown in **Supplementary Figures 1**–**3**, the RDRs of the median, 2.5^th^ and 97.5^th^ percentiles of TSH in the disease-free Yellow subgroup were much lower than those in the disease-free Caucasians group [median: 0.21 (0.03, 0.39) vs 0.44 (0.41, 0.47); 2.5^th^ percentile: 0.13 (0.07, 0.19) vs 0.43 (0.37, 0.49); 97.5^th^ percentile: 0.15 (0.12, 0.18) vs 0.43 (0.41, 0.46)]. Therefore, the corresponding median and 2.5^th^-97.5^th^ percentiles of TSH in disease-free Yellows are generally much higher than those in disease-free Caucasians. As shown in **Table 2**, the RIs of TSH (mU/L) in the Yellow and Caucasian populations are 1.84 (0.58, 6.69) and 1.30 (0.38, 4.49), respectively. The difference between the two ethnicities in the disease-free population is similar to that in the reference population.

### Iodine Status-Specific RIs of TSH in the Disease-Free Subgroups

Compared with the corresponding RIs in different iodine status subgroups in the reference population, the RIs for TSH in the disease-free population were somewhat similar but also had some differences. **Supplementary Figure 4** shows a slightly lower RDR of median TSH in both the iodine-deficient [0.33 (0.19, 0.46)] and more than adequate iodine [0.27 (0.24, 0.29)] subgroups when compared with the iodine-sufficient subgroup [0.45 (0.37, 0.54)]. Therefore, the median TSH in the iodine-sufficient disease-free subgroup (1.28 mU/L) was lower than that in the other two subgroups (1.56 mU/L in the iodine-deficient subgroup and 1.71 mU/L in the more than adequate iodine subgroup) (**Table 2**).

However, the 2.5^th^ and 97.5^th^ percentiles yielded different results from those in the reference population. **Supplementary Figure 5** presents the disease-free RDRs of the 2.5^th^ percentile in the iodine-deficient and iodine-sufficient subgroups, showing that the iodine-deficient subgroup tended to have a much higher RDR [0.46 (0.44, 0.48) vs 0.22 (0.15, 0.30)]. In contrast, the RDR of the 97.5^th^ percentile in the iodine-deficient disease-free subgroup [0.39 (0.26, 0.53)] was slightly lower than that in the iodine-sufficient subgroup [0.43 (0.26, 0.60)] (**Supplementary Figure 6**). Accordingly, the 2.5^th^ and 97.5^th^ percentiles of TSH in the disease-free iodine-deficient subgroup were lower (0.36 mU/L vs 0.52 mU/L) and higher (4.80 mU/L vs 4.49 mU/L), respectively, than those in the iodine-sufficient subgroup (**Table 2**).

## Discussion

A previous study confirmed that the upper limit of the TSH RI (7.04 mU/L) was a record high in mainland China based on the NACB standard. As a supplement to the previous study, we calculated a higher upper limit of the RI (7.87 mU/L) and a wider range of the RI based on the disease-free population, which can serve as a reference in future research in mainland China. A subsequent meta-analysis confirmed the significant impact of ethnicity on TSH levels; Yellows had a much higher TSH level than Caucasians in both the reference and disease-free populations. As in the primary studies, the reference 97.5^th^ percentile of TSH increased with increasing iodine sufficiency worldwide, while the 2.5^th^ percentile and median TSH level were the lowest in the iodine-sufficient reference subgroup.

A number of previous studies have explored the prevalence of SCH, and the rates vary widely from 0.5% to 16.7% (4, 45). Such a broad range of prevalence rates is mainly due to the substantial differences in characteristics such as sex, age, iodine status and genetic background in various studies. Although the NACB guidelines recommend that we exclude subjects with a personal, family or medication history associated with thyroid disease and exclude those with positivity for antibodies or a goiter, many studies have shown that the TSH RI is affected by numerous other factors. In addition to the studies mentioned above, another cross-sectional study in the US explored the influence of ethnicity on TSH levels and found that the TSH distribution in Caucasians was shifted to a significantly higher level than that in the Black population, while the distribution in the Black population was similar to that in the Hispanic population (46). In the present meta-analysis, we demonstrated for the first time the obvious difference in TSH RIs between Yellow and Caucasian patients, in both the reference and disease-free populations. However, the iodine status, test kit brand, and age and sex compositions were not entirely consistent across the included studies, resulting in some heterogeneity. Therefore, the above differences need to be verified in additional studies in the future.

In addition to ethnicity, iodine status is another major factor that determines the RI for TSH. Before iodine supplementation policies were promulgated worldwide, iodine deficiency was the main cause of neonatal hypothyroidism (47), and moderate-to-severe iodine deficiency significantly increases the risk of hypothyroidism (48, 49). However, with the continuous promotion of iodine supplementation policies, more than adequate or excess iodine intake might also increase the prevalence of hypothyroidism (50–53). In addition to the results in the reference population in the TIDE study, the KHANES III study also showed a similar result. The 97.5^th^ percentile of the TSH level in the reference population of South Koreans significantly increased from 4.85 mU/L in the group with a UIC <50 μg/L to 8.74 mU/L in the group with a UIC ≥1000 μg/L (54). Moreover, a shift to the right in the TSH distribution was also observed in the reference population after ten years of iodine supplementation in Germany (30). Based on the above studies, we find that iodine deficiency, more than adequate or excess iodine, is a risk factor for hyperthyrotropinemia, and exploring the ideal range of iodine supplementation is vital for the control and prevention of hypothyroidism.

The results of the present meta-analysis are similar to the above findings. Based on the NACB criteria, we found that the 2.5^th^ percentile and median TSH were the lowest in the iodine-sufficient subgroup. The results indicate that iodine sufficiency is probably a reasonable target for iodine supplementation. However, among subjects with relatively higher TSH levels, the three subgroups with different iodine statuses had distribution patterns that differed slightly from the above results. The 97.5^th^ percentile of TSH had an upward trend with increasing iodine sufficiency. This result is similar to the previous results in our primary study, which confirmed that the 97.5^th^ percentile of the TSH level significantly increased by 0.0132 mU/L with every 10 μg/L increase in the UIC^12^. We speculate that although iodine sufficiency is a proper target for iodine supplementation, the reference population in iodine-sufficient subgroup were not the least affected by hyperthyrotropinemia. The above results might be explained by the exclusion of a number of patients with unknown iodine deficiency-related hypothyroidism when subjects with a palpable or visible goiter were excluded. The above factors probably led to fewer subjects with high levels of TSH in the iodine-deficient group. On the other hand, we did not find similar differences based on the disease-free criteria. We believe that since the exclusion criteria for the disease-free population were not as strict as those in the NACB, a number of subjects with unknown thyroid diseases were included in the analysis. The diseases definitely affect TSH, and having patients with iodine deficiency-related hypothyroidism in the final population would certainly affect the findings. Therefore, the median and 97.5^th^ percentile in the iodine-deficient group were both higher than those in the iodine-sufficient group, while the lower 2.5^th^ percentile in the iodine-deficient group was probably due to the combined effect of the increased median and 97.5^th^ percentile. Similarly, it needs to be emphasized that the results of the meta-analysis could have been impacted by different ethnic backgrounds even in the same iodine status subgroup. The heterogeneity test showed that the studies included in each subgroup were highly heterogeneous; thus, the above findings need to be confirmed in future studies.

The limitations of this study are as follows. First, due to the characteristics of meta-analysis, we could not investigate whether the RDRs between subgroups were significantly different; thus, we could only observe the general trend in RIs. Second, although the studies selected in the meta-analysis were conducted in the regions with clear ethnicities, we cannot ensure that the ethnicity of each participant absolutely meets the requirement. Third, spot UIC tests or the authors’ statements were used to assess the iodine status in the included studies. The duration of iodine supplementation was ignored. Therefore, the long-term effect of iodine supplementation on the RI of TSH still needs investigation in the future.

In conclusion, Yellow individuals have a much higher TSH level than Caucasians in both the reference and disease-free populations. In the reference population, the 2.5^th^ percentile and median TSH in the iodine-sufficient population were the lowest among the subgroups with different iodine statuses, and the 97.5^th^ percentile showed an increasing trend with increasing iodine sufficiency.

## Data Availability Statement

The raw data supporting the TIDE study will be made available by the authors, without undue reservation. The selected studies supporting the meta-analysis were obtained from public databases (i.e., PubMed, EMBASE, Cochrane Library databases and Google Scholar) (12, 19, 20, 24–44), and the detailed information can be found in the Reference.

## Ethics Statement

The studies involving human participants were reviewed and approved by Ethics Committee of China Medical University. The patients/participants provided their written informed consent to participate in this study.

## Author Contributions

XW and ZS: Conceived and designed the meta-analysis. XW, HW, XZ, XG, FZ and YL: Searched and screened the relevant studies. XW, SL, XG and FZ: Estimated the quality of the studies. WT and ZS: Responsible for the nationwide cross-sectional study. XW, YL, HW and XZ: Responsible for the data analysis and manuscript writing. All authors contributed to the article and approved the submitted version.

## Funding

This work was supported by the National Natural Science Foundation of China (Grant No. 81970682).

## Conflict of Interest

The authors declare that the research was conducted in the absence of any commercial or financial relationships that could be construed as a potential conflict of interest.

## References

[1] Biondi B Cappola AR Cooper DS. Subclinical Hypothyroidism: A Review. JAMA (2019) 322(2):153–60. 10.1001/jama.2019.9052 31287527

[2] Hollowell JG Staehling NW Flanders WD Hannon WH Gunter EW Spencer CA. T(4), and thyroid antibodies in the United States population (1988 to 1994): National Health and Nutrition Examination Survey (NHANES III). J Clin Endocrinol Metab (2002) 87(2):489–99. 10.1210/jcem.87.2.8182 11836274

[3] Li Y Teng D Ba J Chen B Du J He L. Efficacy and Safety of Long-Term Universal Salt Iodization on Thyroid Disorders: Epidemiological Evidence from 31 Provinces of Mainland China. Thyroid (2020) 30(4):568–79. 10.1089/thy.2019.0067 32075540

[4] Shan Z Chen L Lian X Liu C Shi B Shi L. Iodine Status and Prevalence of Thyroid Disorders After Introduction of Mandatory Universal Salt Iodization for 16 Years in China: A Cross-Sectional Study in 10 Cities. Thyroid (2016) 26(8):1125–30. 10.1089/thy.2015.0613 27370068

[5] Delitala AP Fanciulli G Maioli M Delitala G. Subclinical hypothyroidism, lipid metabolism and cardiovascular disease. Eur J Intern Med (2017) 38:17–24. 10.1016/j.ejim.2016.12.015 28040402

[6] Moon S Kim MJ Yu JM Yoo HJ Park YJ. Subclinical Hypothyroidism and the Risk of Cardiovascular Disease and All-Cause Mortality: A Meta-Analysis of Prospective Cohort Studies. Thyroid (2018) 28(9):1101–10. 10.1089/thy.2017.0414 29978767

[7] Parle JV Franklyn JA Cross KW Jones SC Sheppard MC. Prevalence and follow-up of abnormal thyrotrophin (TSH) concentrations in the elderly in the United Kingdom. Clin Endocrinol (Oxf) (1991) 34(1):77–83. 10.1111/j.1365-2265.1991.tb01739.x 2004476

[8] Ehrenkranz J Bach PR Snow GL Schneider A Lee JL Ilstrup S. Circadian and Circannual Rhythms in Thyroid Hormones: Determining the TSH and Free T4 Reference Intervals Based Upon Time of Day, Age, and Sex. Thyroid (2015) 25(8):954–61. 10.1089/thy.2014.0589 26061389

[9] Li C Shan Z Mao J Wang W Xie X Zhou W. Assessment of thyroid function during first-trimester pregnancy: what is the rational upper limit of serum TSH during the first trimester in Chinese pregnant women? J Clin Endocrinol Metab (2014) 99(1):73–9. 10.1210/jc.2013-1674 24276458

[10] Reinehr T Isa A de Sousa G Dieffenbach R Andler W. Thyroid hormones and their relation to weight status. Horm Res (2008) 70(1):51–7. 10.1159/000129678 18493150

[11] Zhai X Zhang L Chen L Lian X Liu C Shi B. An Age-Specific Serum Thyrotropin Reference Range for the Diagnosis of Thyroid Diseases in Older Adults: A Cross-Sectional Survey in China. Thyroid (2018) 28(12):1571–9. 10.1089/thy.2017.0715 30351201

[12] Zhao L Teng D Shi X Li Y Ba J Chen B. The Effect of Increased Iodine Intake on Serum Thyrotropin: A Cross-Sectional, Chinese Nationwide Study. Thyroid (2020) 30(12):1810–9. 10.1089/thy.2019.0842 32762331

[13] Bagchi N Brown TR Parish RF. Thyroid dysfunction in adults over age 55 years. A study in an urban US community. Arch Intern Med (1990) 150(4):785–7. 10.1001/archinte.150.4.785 2109585

[14] Schectman JM Kallenberg GA Hirsch RP Shumacher RJ. Report of an association between race and thyroid stimulating hormone level. Am J Public Health (1991) 81(4):505–6. 10.2105/ajph.81.4.505 PMC1405055 2003636

[15] Walker JA Illions EH Huddleston JF Smallridge RC. Racial comparisons of thyroid function and autoimmunity during pregnancy and the postpartum period. Obstet Gynecol (2005) 106(6):1365–71. 10.1097/01.AOG.0000185475.61612.ea 16319264

[16] Zhou W Brumpton B Kabil O Gudmundsson J Thorleifsson G Weinstock J. GWAS of thyroid stimulating hormone highlights pleiotropic effects and inverse association with thyroid cancer. Nat Commun (2020) 11(1):3981. 10.1038/s41467-020-17718-z 32769997 PMC7414135

[17] Guan H Shan Z Teng X Li Y Teng D Jin Y. Influence of iodine on the reference interval of TSH and the optimal interval of TSH: results of a follow-up study in areas with different iodine intakes. Clin Endocrinol (Oxf) (2008) 69(1):136–41. 10.1111/j.1365-2265.2007.03150.x 18042176

[18] Baloch Z Carayon P Conte-Devolx B Demers LM Feldt-Rasmussen U Henry JF. Laboratory medicine practice guidelines. Laboratory support for the diagnosis and monitoring of thyroid disease. Thyroid (2003) 13(1):3–126. 10.1089/105072503321086962 12625976

[19] Raverot V Bonjour M Abeillon du Payrat J Perrin P Roucher-Boulez F Lasolle H. Age- and Sex-Specific TSH Upper-Limit Reference Intervals in the General French Population: There Is a Need to Adjust Our Actual Practices. J Clin Med (2020) 9(3):792. 10.3390/jcm9030792 PMC7141356 32183257

[20] Vadiveloo T Donnan PT Murphy MJ Leese GP. Age- and gender-specific TSH reference intervals in people with no obvious thyroid disease in Tayside, Scotland: the Thyroid Epidemiology, Audit, and Research Study (TEARS). J Clin Endocrinol Metab (2013) 98(3):1147–53. 10.1210/jc.2012-3191 23345094

[21] Li Y Teng D Shi X Qin G Qin Y Quan H. Prevalence of diabetes recorded in mainland China using 2018 diagnostic criteria from the American Diabetes Association: national cross sectional study. BMJ (2020) 369:m997. 10.1136/bmj.m997 32345662 PMC7186854

[22] Gao X Li Y Li J Liu A Sun W Teng W. and FT4 Reference Intervals in Chinese Women: A Systematic Review and Meta-Analysis. Front Endocrinol (Lausanne) (2018) 9:432. 10.3389/fendo.2018.00432 30123185 PMC6086137

[23] Stang A. Critical evaluation of the Newcastle-Ottawa scale for the assessment of the quality of nonrandomized studies in meta-analyses. Eur J Epidemiol (2010) 25(9):603–5. 10.1007/s10654-010-9491-z 20652370

[24] Amouzegar A Delshad H Mehran L Tohidi M Khafaji F Azizi F. Reference limit of thyrotropin (TSH) and free thyroxine (FT4) in thyroperoxidase positive and negative subjects: a population based study. J Endocrinol Invest (2013) 36(11):950–4. 10.3275/9033 23873252

[25] Cai J Fang Y Jing D Xu S Ming J Gao B. Reference intervals of thyroid hormones in a previously iodine-deficient but presently more than adequate area of Western China: a population-based survey. Endocr J (2016) 63(4):381–8. 10.1507/endocrj.EJ15-0574 26842591

[26] Chan AO Iu YP Shek CC. The reference interval of thyroid-stimulating hormone in Hong Kong Chinese. J Clin Pathol (2011) 64(5):433–6. 10.1136/jcp.2010.087627 21422036

[27] Clerico A Trenti T Aloe R Dittadi R Rizzardi S Migliardi M. A multicenter study for the evaluation of the reference interval for TSH in Italy (ELAS TSH Italian Study). Clin Chem Lab Med (2018) 57(2):259–67. 10.1515/cclm-2018-0541 30016276

[28] d’Herbomez M Jarrige V Darte C. Reference intervals for serum thyrotropin (TSH) and free thyroxine (FT4) in adults using the Access Immunoassay System. Clin Chem Lab Med (2005) 43(1):102–5. 10.1515/CCLM.2005.017 15653452

[29] Hickman PE Koerbin G Simpson A Potter JM Hughes DG Abhayaratna WP. Using a thyroid disease-free population to define the reference interval for TSH and free T4 on the Abbott Architect analyser. Clin Endocrinol (Oxf) (2017) 86(1):108–12. 10.1111/cen.13143 27333057

[30] Ittermann T Khattak RM Nauck M Cordova CM Volzke H. Shift of the TSH reference range with improved iodine supply in Northeast Germany. Eur J Endocrinol (2015) 172(3):261–7. 10.1530/EJE-14-0898 25452467

[31] Jensen E Hyltoft Petersen P Blaabjerg O Hansen PS Brix TH Kyvik KO. Establishment of a serum thyroid stimulating hormone (TSH) reference interval in healthy adults. The importance of environmental factors, including thyroid antibodies. Clin Chem Lab Med (2004) 42(7):824–32. 10.1515/CCLM.2004.136 15327019

[32] Kahapola-Arachchige KM Hadlow N Wardrop R Lim EM Walsh JP. Age-specific TSH reference ranges have minimal impact on the diagnosis of thyroid dysfunction. Clin Endocrinol (Oxf) (2012) 77(5):773–9. 10.1111/j.1365-2265.2012.04463.x 22703566

[33] Kim M Kim TY Kim SH Lee Y Park SY Kim HD. Reference interval for thyrotropin in a ultrasonography screened Korean population. Korean J Intern Med (2015) 30(3):335–44. 10.3904/kjim.2015.30.3.335 PMC4438288 25995664

[34] Kratzsch J Fiedler GM Leichtle A Brugel M Buchbinder S Otto L. New reference intervals for thyrotropin and thyroid hormones based on National Academy of Clinical Biochemistry criteria and regular ultrasonography of the thyroid. Clin Chem (2005) 51(8):1480–6. 10.1373/clinchem.2004.047399 15961550

[35] Kutluturk F Yildirim B Ozturk B Ozyurt H Bekar U Sahin S. The reference intervals of thyroid stimulating hormone in healthy individuals with normal levels of serum free thyroxine and without sonographic pathologies. Endocr Res (2014) 39(2):56–60. 10.3109/07435800.2013.824896 24067097

[36] Li C Guan H Teng X Lai Y Chen Y Yu J. An epidemiological study of the serum thyrotropin reference range and factors that influence serum thyrotropin levels in iodine sufficient areas of China. Endocr J (2011) 58(11):995–1002. 10.1507/endocrj.k11e-101 21959332

[37] Marwaha RK Tandon N Ganie MA Mehan N Sastry A Garg MK. Reference range of thyroid function (FT3, FT4 and TSH) among Indian adults. Clin Biochem (2013) 46(4-5):341–5. 10.1016/j.clinbiochem.2012.09.021 23041244

[38] Mirjanic-Azaric B Avram S Stojakovic-Jelisavac T Stojanovic D Petkovic M Bogavac-Stanojevic N. Direct Estimation of Reference Intervals for Thyroid Parameters in the Republic of Srpska. J Med Biochem (2017) 36(2):137–44. 10.1515/jomb-2017-0008 PMC5471646 28680357

[39] O’Leary PC Feddema PH Michelangeli VP Leedman PJ Chew GT Knuiman M. Investigations of thyroid hormones and antibodies based on a community health survey: the Busselton thyroid study. Clin Endocrinol (Oxf) (2006) 64(1):97–104. 10.1111/j.1365-2265.2005.02424.x 16402936

[40] Quinn FA Tam MC Wong PT Poon PK Leung MS. Thyroid autoimmunity and thyroid hormone reference intervals in apparently healthy Chinese adults. Clin Chim Acta (2009) 405(1-2):156–9. 10.1016/j.cca.2009.04.021 19410568

[41] Ren B Wan S Liu L Qu M Wu H Shen H. Distributions of serum thyroid-stimulating hormone in 2020 thyroid disease-free adults from areas with different iodine levels: a cross-sectional survey in China. J Endocrinol Invest (2020). 10.1007/s40618-020-01395-2 32816248

[42] Takeda K Mishiba M Sugiura H Nakajima A Kohama M Hiramatsu S. Evaluated reference intervals for serum free thyroxine and thyrotropin using the conventional outliner rejection test without regard to presence of thyroid antibodies and prevalence of thyroid dysfunction in Japanese subjects. Endocr J (2009) 56(9):1059–66. 10.1507/endocrj.k09e-123 19738362

[43] Tozzoli R D’Aurizio F Metus P Steffan A Mazzon C Bagnasco M. Reference intervals for thyrotropin in an area of Northern Italy: the Pordenone thyroid study (TRIPP). J Endocrinol Invest (2018) 41(8):985–94. 10.1007/s40618-018-0825-0 29340973

[44] Volzke H Alte D Kohlmann T Ludemann J Nauck M John U. Reference intervals of serum thyroid function tests in a previously iodine-deficient area. Thyroid (2005) 15(3):279–85. 10.1089/thy.2005.15.279 15785248

[45] Delshad H Mehran L Tohidi M Assadi M Azizi F. The incidence of thyroid function abnormalities and natural course of subclinical thyroid disorders, Tehran, I.R. Iran. J Endocrinol Invest (2012) 35(5):516–21. 10.3275/7968 21971483

[46] Boucai L Surks MI. Reference limits of serum TSH and free T4 are significantly influenced by race and age in an urban outpatient medical practice. Clin Endocrinol (Oxf) (2009) 70(5):788–93. 10.1111/j.1365-2265.2008.03390.x 18727705

[47] Roberts CG Ladenson PW. Hypothyroidism. Lancet (2004) 363(9411):793–803. 10.1016/S0140-6736(04)15696-1 15016491

[48] Zimmermann MB. Iodine deficiency. Endocr Rev (2009) 30(4):376–408. 10.1210/er.2009-0011 19460960

[49] Zimmermann MB Jooste PL Pandav CS. Iodine-deficiency disorders. Lancet (2008) 372(9645):1251–62. 10.1016/S0140-6736(08)61005-3 18676011

[50] Aghini Lombardi F Fiore E Tonacchera M Antonangeli L Rago T Frigeri M. The effect of voluntary iodine prophylaxis in a small rural community: the Pescopagano survey 15 years later. J Clin Endocrinol Metab (2013) 98(3):1031–9. 10.1210/jc.2012-2960 23436921

[51] Petersen M Knudsen N Carle A Andersen S Jorgensen T Perrild H. Increased Incidence Rate of Hypothyroidism After Iodine Fortification in Denmark: A 20-Year Prospective Population-Based Study. J Clin Endocrinol Metab (2019) 104(5):1833–40. 10.1210/jc.2018-01993 30551165

[52] Teng W Shan Z Teng X Guan H Li Y Teng D. Effect of iodine intake on thyroid diseases in China. N Engl J Med (2006) 354(26):2783–93. 10.1056/NEJMoa054022 16807415

[53] Teng X Shan Z Chen Y Lai Y Yu J Shan L. More than adequate iodine intake may increase subclinical hypothyroidism and autoimmune thyroiditis: a cross-sectional study based on two Chinese communities with different iodine intake levels. Eur J Endocrinol (2011) 164(6):943–50. 10.1530/EJE-10-1041 21444648

[54] Jeon MJ Kim WG Kwon H Kim M Park S Oh HS. Excessive Iodine Intake and Thyrotropin Reference Interval: Data from the Korean National Health and Nutrition Examination Survey. Thyroid (2017) 27(7):967–72. 10.1089/thy.2017.0078 28471294

