# Supplementary figures and images for: Reference Intervals for Serum Thyroid-Stimulating Hormone Based on a Recent Nationwide Cross-Sectional Study and Meta-Analysis
Source: Front Endocrinol (Lausanne). 2021 Jun 1;12:660277. doi: 10.3389/fendo.2021.660277 (PMC8204855; doi:10.3389/fendo.2021.660277)

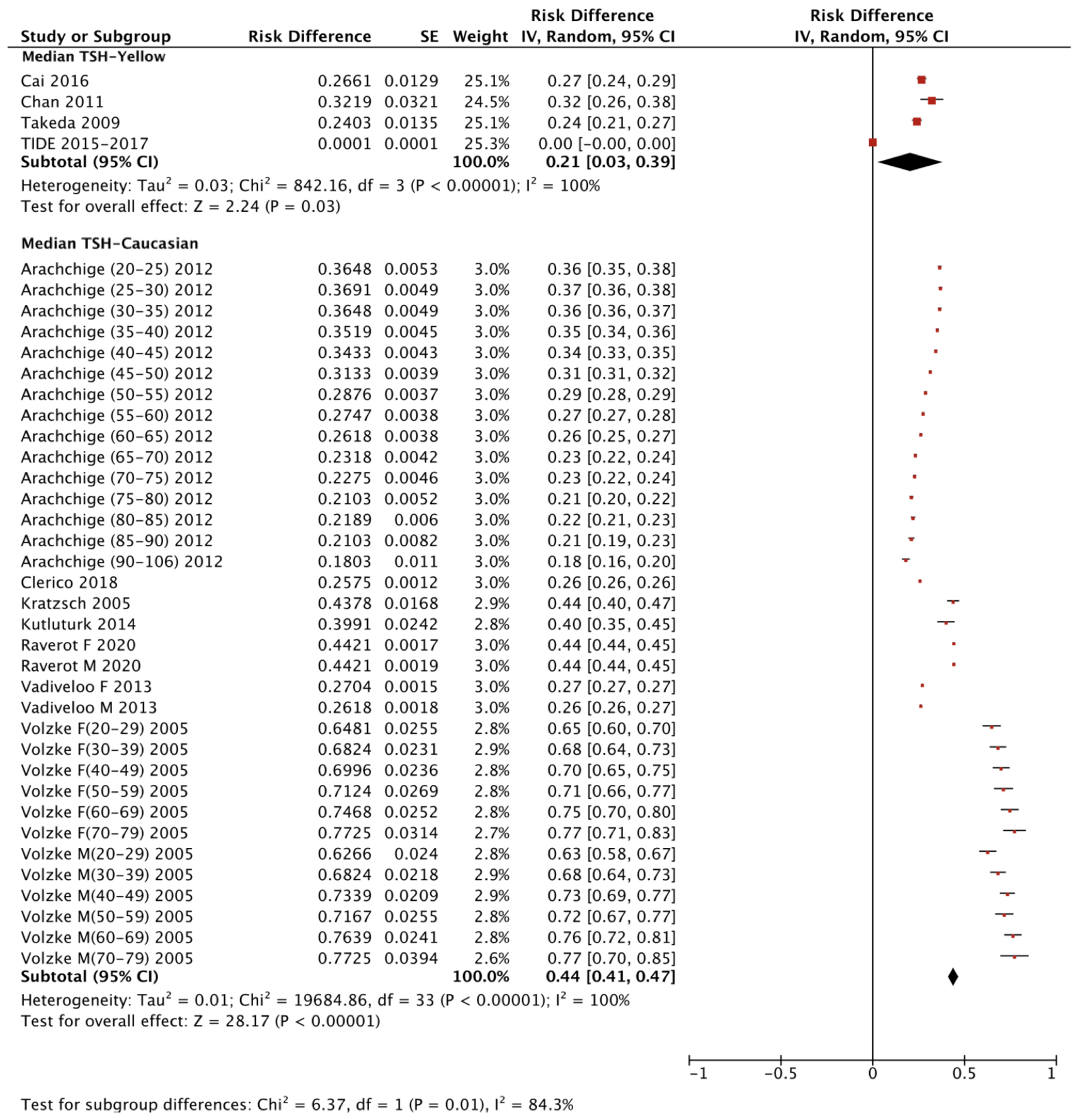

Supplement: Supplementary file 1 [file Image_1.jpeg]

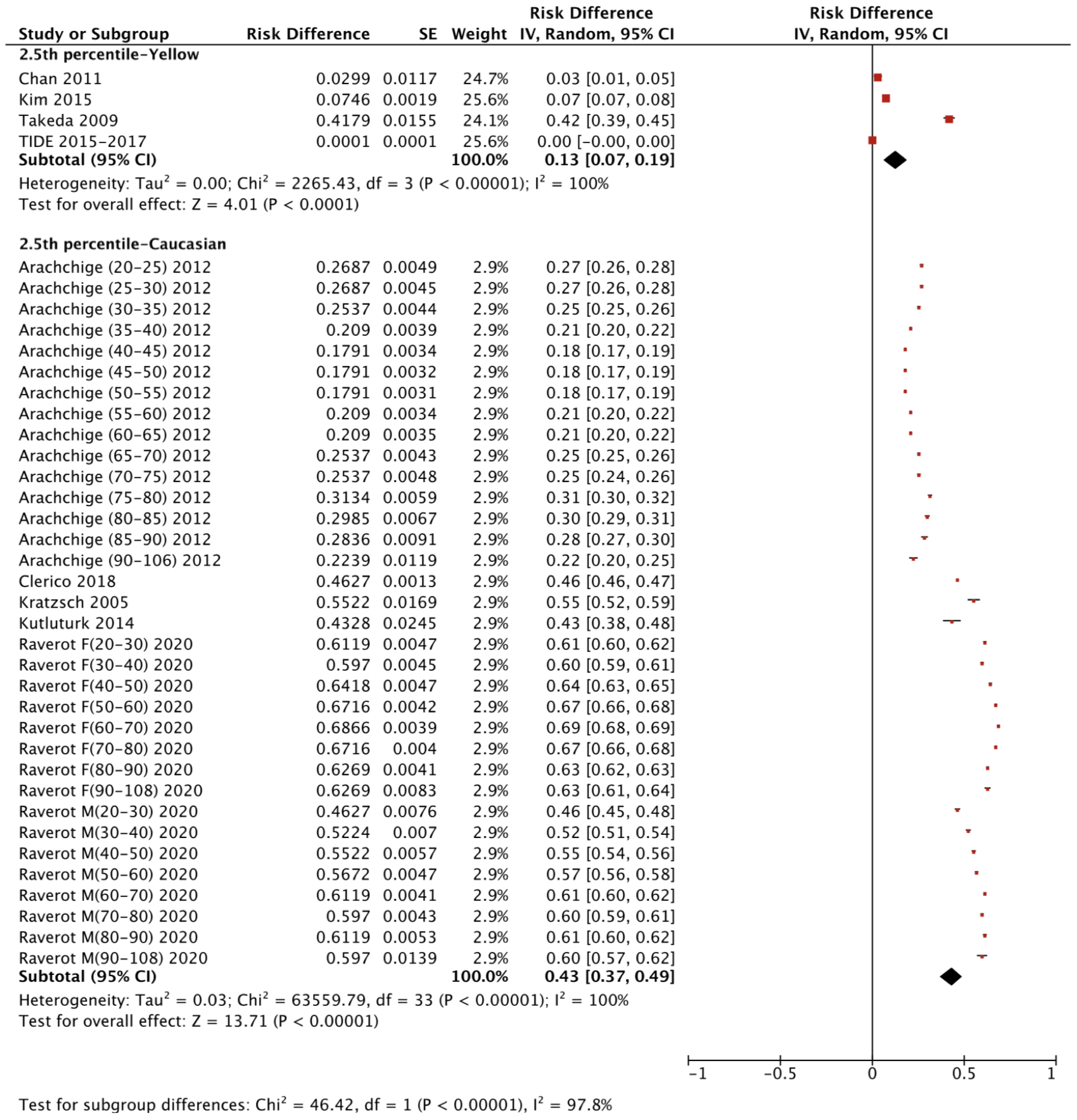

Supplement: Supplementary file 2 [file Image_2.jpeg]

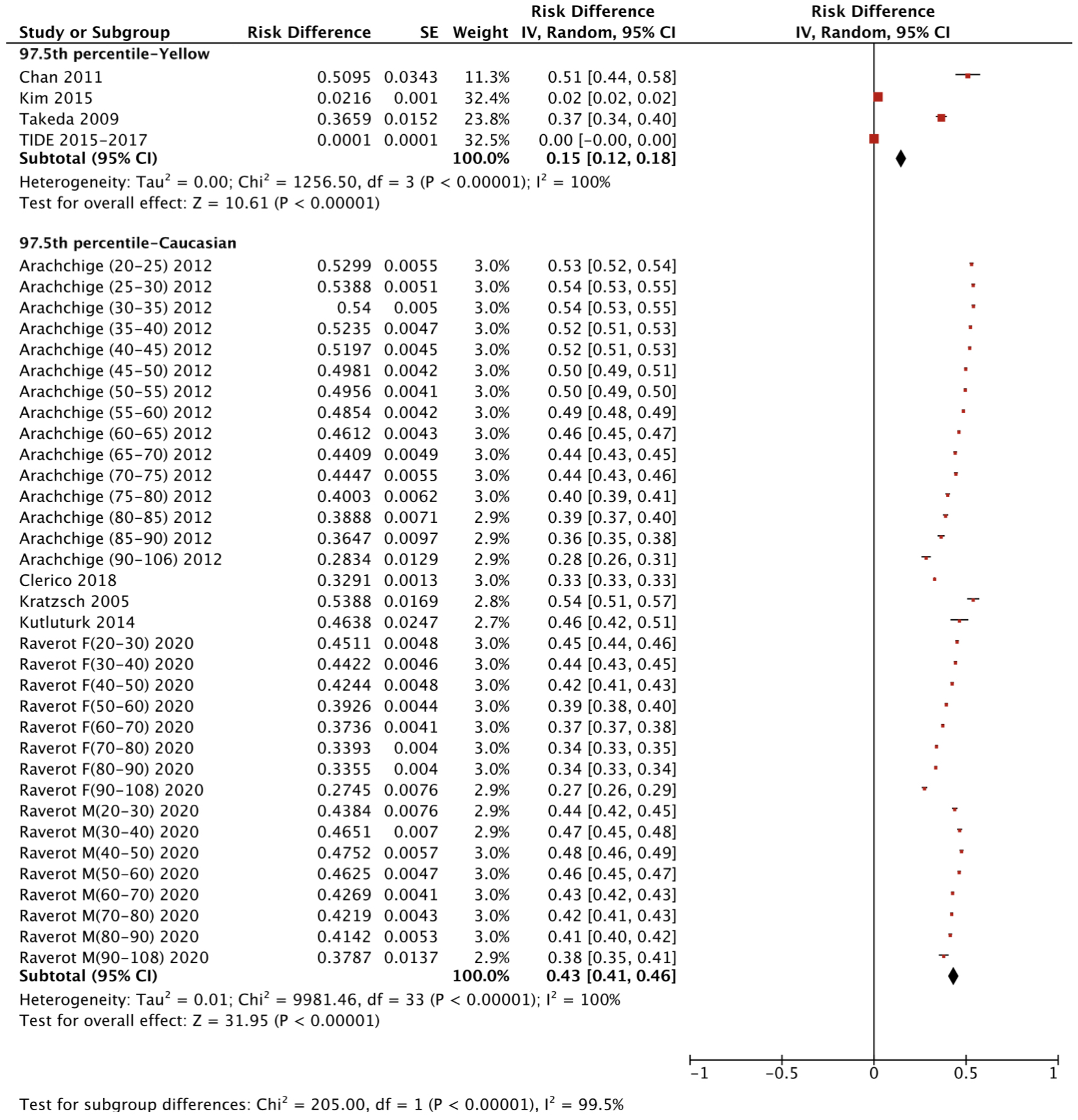

Supplement: Supplementary file 3 [file Image_3.jpeg]

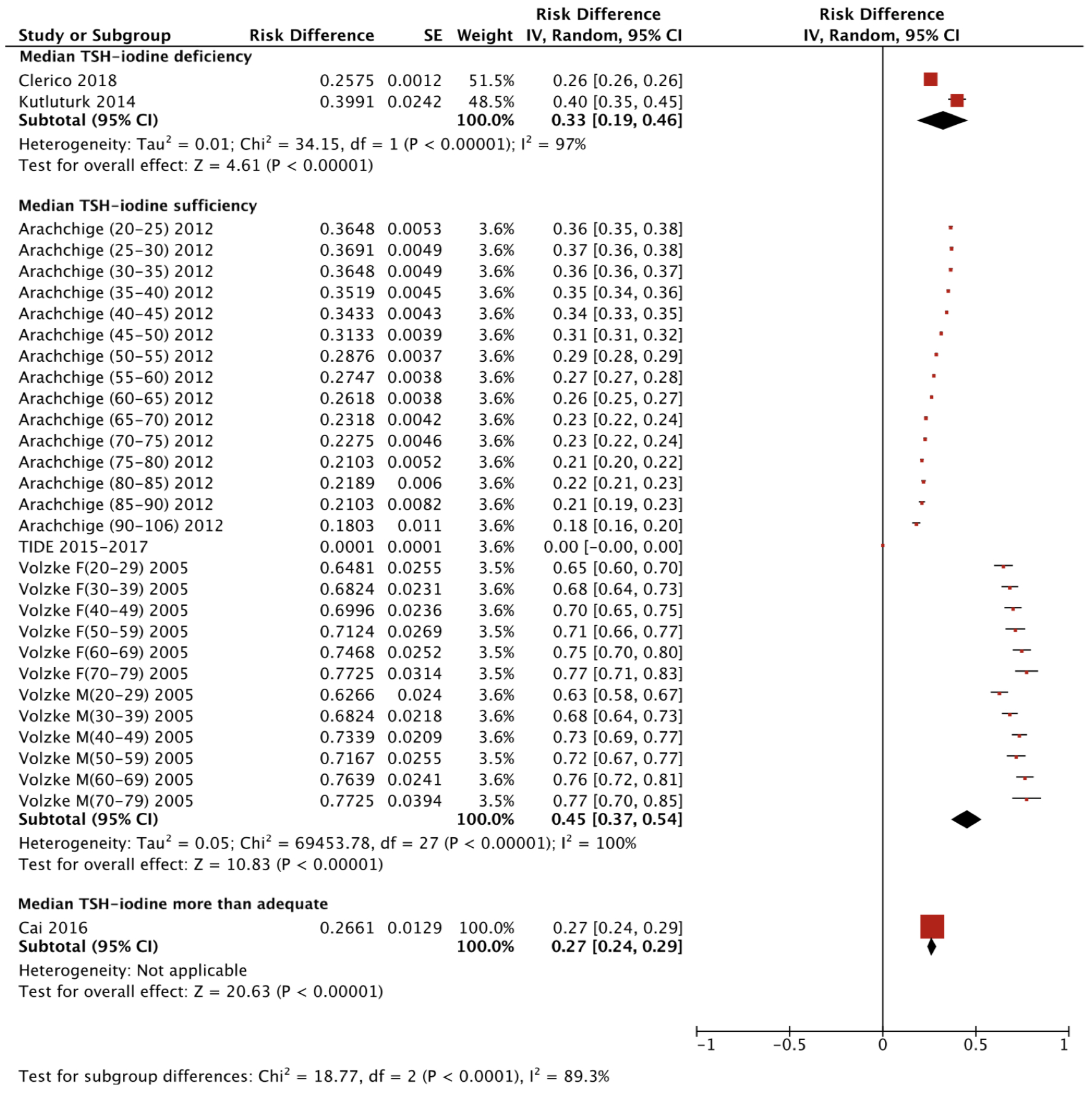

Supplement: Supplementary file 4 [file Image_4.jpeg]

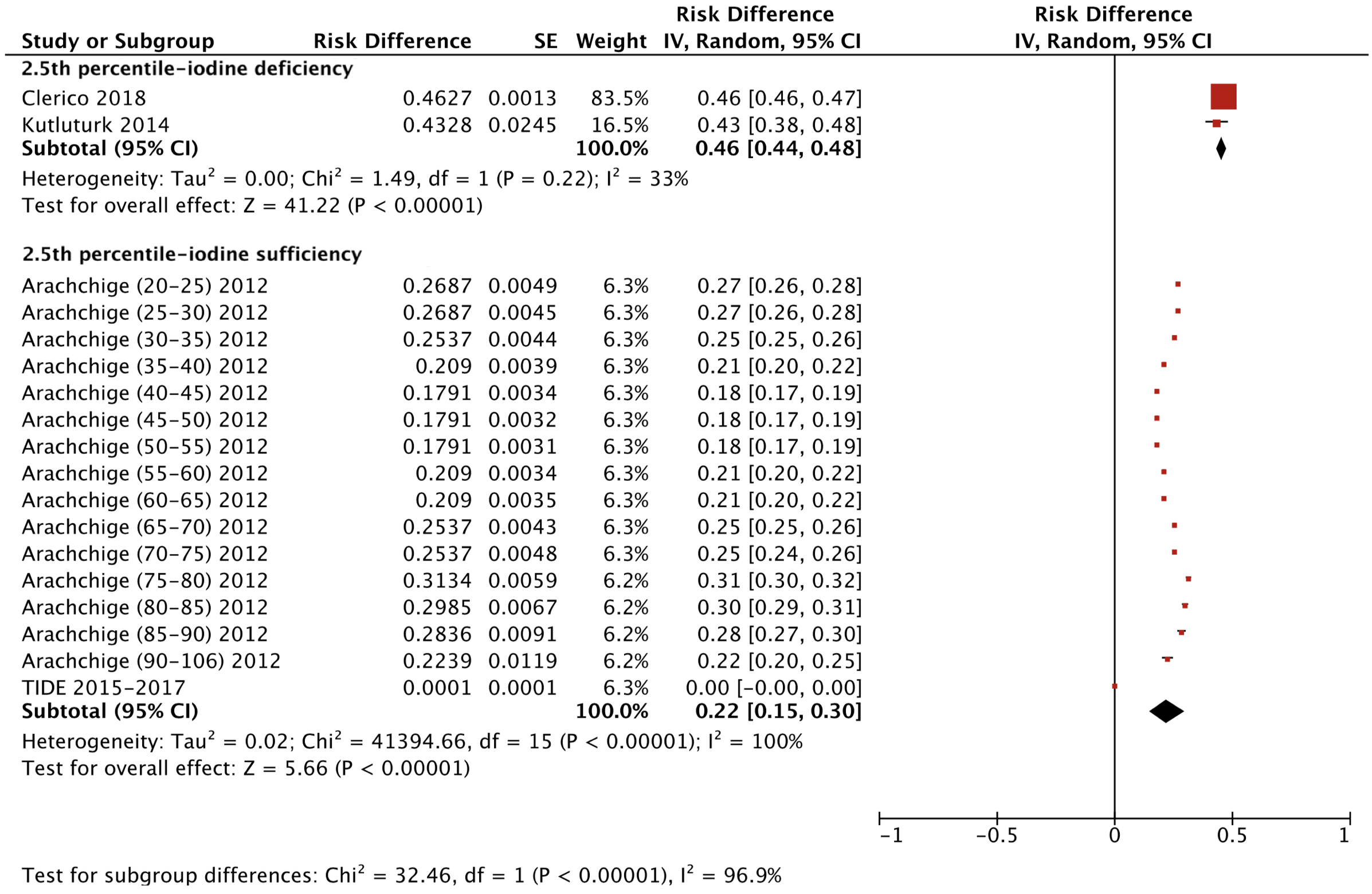

Supplement: Supplementary file 5 [file Image_5.jpeg]

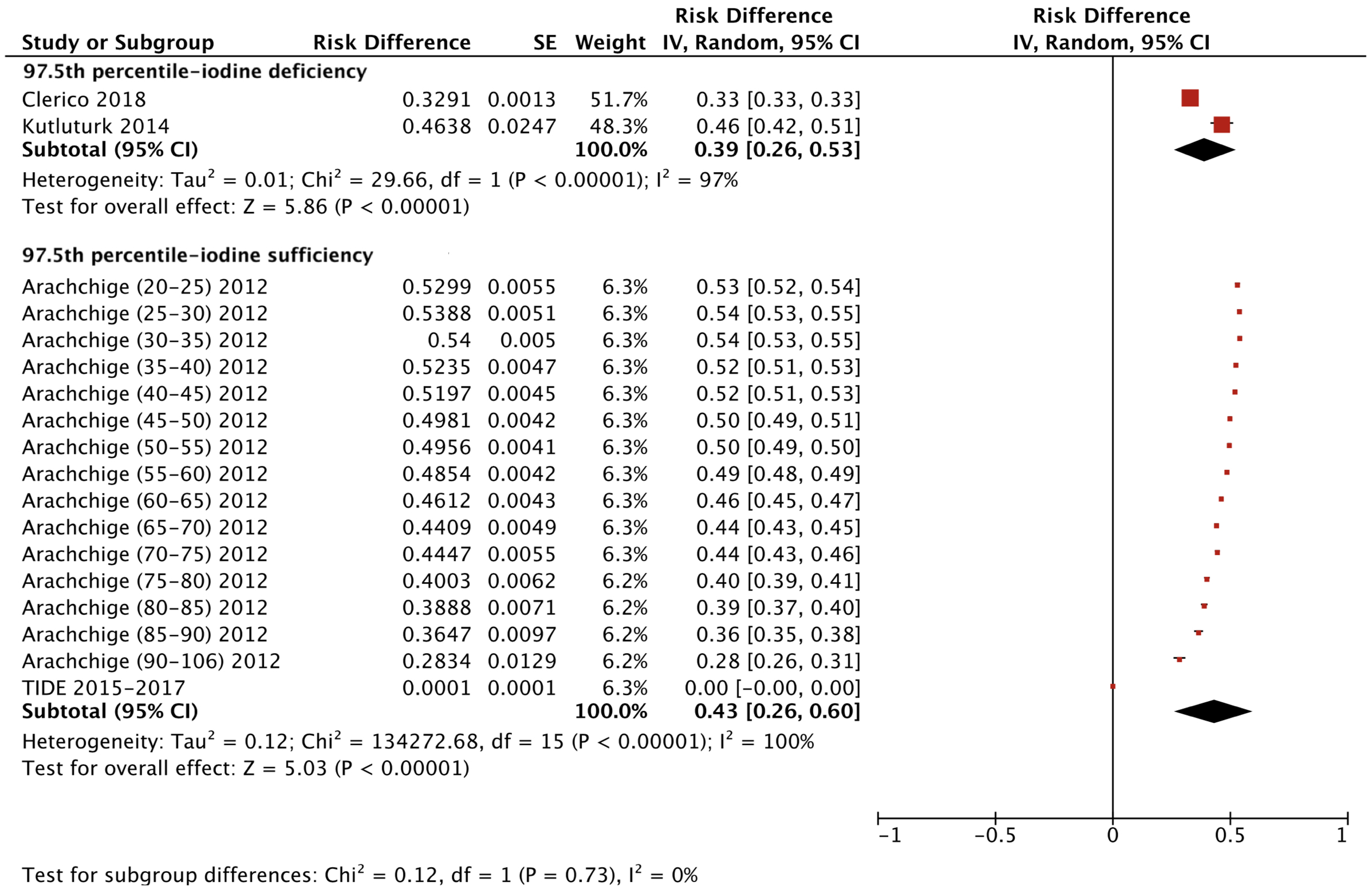

Supplement: Supplementary file 6 [file Image_6.jpeg]
