# Supplementary material for: Reference Intervals for Serum Thyroid-Stimulating Hormone Based on a Recent Nationwide Cross-Sectional Study and Meta-Analysis
Source: Front Endocrinol (Lausanne). 2021 Jun 1;12:660277. doi: 10.3389/fendo.2021.660277 (PMC8204855; doi:10.3389/fendo.2021.660277)
Supplement: Supplementary file 7 [file DataSheet_1.docx]

Supplementary Material

**Supplementary Figure 1. Ethnicity standardized forest plots of the pooled relative descent rate (%) of median TSH (50^th^ percentile) in disease-free populations.**

The numbers in parenthesis represent the age range of the corresponding subgroup.

**Supplementary Figure 2. Ethnicity standardized forest plots of the pooled relative descent rate (%) of TSH lower limits (2.5^th^ percentile) in disease-free populations.**

**Supplementary Figure 3. Ethnicity standardized forest plots of the pooled relative descent rate (%) of TSH upper limits (97.5^th^ percentile) in disease-free populations.**

**Supplementary Figure 4. Iodine status standardized forest plots of the pooled relative descent rate (%) of median TSH (50^th^ percentile) in disease-free populations.**

**Supplementary Figure 5. Iodine status standardized forest plots of the pooled relative descent rate (%) of TSH lower limits (2.5^th^ percentile) in disease-free populations.**

**Supplementary Figure 6. Iodine status standardized forest plots of the pooled relative descent rate (%) of TSH upper limits (97.5^th^ percentile) in disease-free populations.**
